# A New Role For Green Leaf Volatile Esters in Tomato Stomatal Defense Against *Pseudomonas syringe* pv. *tomato*

**DOI:** 10.3389/fpls.2018.01855

**Published:** 2018-12-18

**Authors:** María Pilar López-Gresa, Celia Payá, Miguel Ozáez, Ismael Rodrigo, Vicente Conejero, Harry Klee, José María Bellés, Purificación Lisón

**Affiliations:** ^1^Instituto de Biología Molecular y Celular de Plantas, Universitat Politècnica de València-Consejo Superior de Investigaciones Científicas, Valencia, Spain; ^2^Horticultural Sciences Department, University of Florida, Gainesville, FL, United States

**Keywords:** AAT1, GLV esters, stomata, defense, tomato, bacteria

## Abstract

The volatile esters of (Z)-3-hexenol with acetic, propionic, isobutyric, or butyric acids are synthesized by alcohol acyltransferases (AAT) in plants. These compounds are differentially emitted when tomato plants are efficiently resisting an infection with *Pseudomonas syringae* pv. *tomato*. We have studied the defensive role of these green leaf volatile (GLV) esters in the tomato response to bacterial infection, by analyzing the induction of resistance mediated by these GLVs and the phenotype upon bacterial infection of tomato plants impaired in their biosynthesis. We observed that treatments of plants with (Z)-3-hexenyl propionate (HP) and, to a greater extent with (Z)-3-hexenyl butyrate (HB), resulted in stomatal closure, PR gene induction and enhanced resistance to the bacteria. HB-mediated stomatal closure was also effective in several plant species belonging to *Nicotiana, Arabidopsis, Medicago, Zea* and *Citrus* genus, and both stomatal closure and resistance were induced in HB-treated *NahG* tomato plants, which are deficient in salicylic acid (SA) accumulation. Transgenic antisense *AAT1* tomato plants, which displayed a reduction of ester emissions upon bacterial infection in leaves, exhibited a lower ratio of stomatal closure and were hyper-susceptible to bacterial infection. Our results confirm the role of GLV esters in plant immunity, uncovering a SA-independent effect of HB in stomatal defense. Moreover, we identified HB as a natural stomatal closure compound with potential agricultural applications.

## Introduction

Plants synthesize different defensive secondary metabolites in response to biotic stresses. These compounds may exert direct defensive functions by acting on the pathogen, or act indirectly by activating the defensive response of the plant. Some volatile organic compounds (VOCs) belong to this group of defensive molecules. These VOCs are typically lipophilic liquids with high vapor pressures that can freely cross membranes and be emitted (Dudareva et al., 2006).

The defensive role of VOCs has been classically associated with plant defense against herbivores. Emitted volatiles can directly affect herbivores due to their toxic, repelling or deterring properties (Kessler and Baldwin, 2001; Aharoni et al., 2003). They can also attract predators of the attacking insects, thus displaying tritrophic interactions (Drukker et al., 2000; Kessler and Baldwin, 2001). VOCs can also prime defenses in neighboring plants, constituting mechanisms of interplant communication (Kim and Felton, 2013). Although the roles of VOCs in pathogen defense have received less attention, some volatiles have been implicated in different plant-pathogen interactions (Croft et al., 1993; Huang et al., 2003; Cardoza and Tumlinson, 2006; Niinemets et al., 2013; Hijaz et al., 2016; Ameye et al., 2018).

Recently, a GC-MS metabolomic analysis revealed a number of VOCs that are differentially emitted by tomato cv. Rio Grande plants containing the *Pto* gene, upon infection with virulent or avirulent strains of the bacterium *Pseudomonas syringae* DC3000 pv. *tomato* (*Pst*) (López-Gresa et al., 2017). Infection of this cultivar with avirulent bacteria, carrying both AvrPto and AvrPtoB effectors, elicits establishment of Effector Triggered Immunity (ETI) in the plant, thus preventing development of the infection (Dangl and Jones, 2001; Lin and Martin, 2005). Metabolomic analysis showed that esters of (*Z*)-3-hexenol with acetic (HA), propionic (HP), isobutyric (HiB) or butyric (HB) acids were differentially emitted during ETI (López-Gresa et al., 2017).

These esters belong to the family of green leaf volatiles (GLVs) that are generated through the oxylipin pathway from C18 polyunsaturated fatty acids (PUFAs), such as linolenic or linoleic acids. The first step in their biosynthesis is peroxidation of the PUFAs performed by the action of lipoxygenases (LOXs). These enzymes can act upon PUFAs at either the C9 or C13 position, being classified as 9-LOX and 13-LOX, respectively. GLV esters are known to be synthesized by 13-LOX via 13-hydroperoxides that are later cleaved by 13-hydroperoxide lyases (13-HPL) into (*Z*)-3-hexenal. This aldehyde is reduced by an alcohol dehydrogenase (ADH), and alcohol acyltransferases (AAT) catalyze esterification of an acyl moiety from an acyl-coenzyme A (acyl-CoA) donor onto an alcohol (Scala et al., 2013; Ameye et al., 2018).

Five alcohol acyltransferase genes (*SlAAT1-5*) have been identified in tomato (Goulet et al., 2015). To confirm the function of this protein, tomato plants containing an *AAT1* antisense construct (*as-AAT1*) were generated, resulting in transgenic fruits with reduced acetate ester emissions (Goulet et al., 2015). Consistent with this biochemical activity and the metabolomic data, tomato plants triggering ETI and differentially emitting these GLV esters, significantly induced *AAT1* expression (López-Gresa et al., 2017). These results suggest a possible role of this enzyme and its products in plant defense.

Many foliar pathogens, including bacteria, primarily invade plants through natural surface opening such as wounds or stomata. Plants have developed the ability to restrict entry of pathogens by closing stomata or by inhibition of stomatal opening (Arnaud and Hwang, 2015). Specifically, *Pst* has been observed to activate plant stomatal closure. To evade this innate immune response, *Pst* has evolved specific virulence factors, including coronatine, to effectively cause stomatal reopening. Coronatine is a mimic of the active JA-Ile hormone, and acts as a suppressor of stomata closing by inhibiting SA-induced closing of stomata. Abscisic acid (ABA) and salicylic acid (SA) have emerged as positive regulators of stomatal defense, while jasmonic acid (JA) is a negative regulator (Melotto et al., 2006, 2008) reviewed in Panchal and Melotto (2017). Although stomata closing is regulated by master ABA and SA signals, it is becoming clear that during biotic interactions, SA is produced upon *Pst* sensing, triggering stomata closing. This SA-induced stomata closing is hijacked by the COR phytotoxin, through the antagonistic the SA-JA crosstalk (Zheng et al., 2012; Gimenez-Ibanez et al., 2017).

To study the possible defensive role of the GLV esters differentially emitted by tomato plants resisting bacterial infection we used a pharmacological approach, assessing the induction of resistance mediated by exogenous treatment with these volatiles. We also examined the mode of action of these compounds and the possible activation of the salicylic acid (SA) pathway caused by VOC treatments. To confirm the defensive role of VOCs, we also used a genetic approach by studying the phenotype of *as-AAT1* tomato plants upon bacterial infection. Our results confirm the role of GLV esters in plant immunity, uncovering a new function of VOCs in stomatal defense. The effect on stomatal aperture of one of these GLV esters was also studied in several plant species, defining a potential use in agriculture.

## Materials and Methods

### Plant Material and GLV Treatments

We used in this study the following tomato plants: *NahG* (Brading et al., 2000) and their corresponding parental Moneymaker plants; *as-AAT1* (Goulet et al., 2015) and Flora-Dade (FD) parental plants; and Rio Grande plants, the only variety containing the *Pto* resistance gene.

The seeds were sterilized with a 1:1 mixture of commercial sodium hypochlorite and distilled H_2_O containing a few drops of Tween 20, and then they were sequentially washed with distilled H_2_O during 5, 10, and 15 min, respectively. After sterilization treatments, seeds were placed in 12 cm-diameter pots that contained a 1:1 mixture of vermiculite and peat and grown under greenhouse conditions with a relative humidity between 50 and 70% and a 16/8 h (26/30°C) light/dark photoperiod.

Four-week-old tomato plants were treated with (*Z*)-3-hexenyl acetate (HA), (*Z*)-3-hexenyl propionate (HP), (*Z*)-3-hexenyl butyrate (HB), and (*Z*)-3-hexenyl isobutyrate (HiB) into 110 L methacrylate chambers. A concentration of 5 μM of each compound was placed in different hydrophilic cotton buds, and distilled H_2_O in the case of the control plants. To avoid VOC mixtures, the treatments were performed individually. Methacrylate chambers were hermetically sealed during the 24 h treatment.

### Bacterial Strain, Bacterial Inoculation and CFU Determination

The bacterial strains used in this study were *Pst* DC3000, and *Pst* DC3000 containing deletions in genes *avrPto* and *avrPtoB* (*Pst* DC3000 Δ*avrPto*/Δ*avrPtoB*) (Lin and Martin, 2005; Ntoukakis et al., 2009). Bacteria were grown during 48 h at 28°C in LB agar medium with different antibiotics: rifampicin (10 mg/mL) and kanamicin (0.5 mg/mL) for *Pst* DC3000, and rifampicin (10 mg/mL), kanamycin (0.25 mg/mL), and spectinomycin (2.5 mg/mL) for *Pst* DC3000 Δ*avrPto*/Δ*avrPtoB*. When colonies were grown, they were transferred into 3 mL of King's B liquid medium supplemented with rifampicin and were grown overnight at 28°C under continuous stirring. After 24 h, colonies were transferred into 14 mL of King's B liquid medium and were grown under the same conditions than the previous step. Then, bacteria were centrifugated 15 min at 3,000 rpm and resuspended in 10 mM sterile MgCl_2_ to an optical density of 0.1 at 600 nm, which corresponds to a final inoculum concentration of 5 × 10^7^ CFU/mL approximately.

Inoculation with bacteria was carried out in 4-week-old tomato plants by immersion or injection methods. For immersion experiments tomato plants were dipped into the bacterial suspension containing 0.05% Silwet L-77. To carry out bacterial injection experiments, each leaflet of the third and fourth leaves was inoculated with a needless syringe by injecting the bacterial suspension into different sites of the leaflet's abaxial side.

For determining bacterial growth, three leaf disks (1 cm^2^ each) were sampled from the bacterial infected leaves from each plant. A total of five plants were used per treatment or tomato plant variety. Density of bacterial populations was determined by plating serial dilutions of the infected material on King's B medium containing rifampicin and counting colony growth.

### Stomatal Aperture Measurement

Tomato leaf impressions were obtained by applying a thin layer of nitrocellulose-based glue (Imedio, Bolton Group, Madrid, Spain) in the abaxial part of the leaves, and then peeling off the glue carefully. In order to observe the stomata, epidermis peels were placed on glass slices and observed under a Leica DC5000 microscope (Leica Microsystems S.L.U.). Pictures of different regions of tomato leaves were taken and at least 50 stomata of each plant and/or each treatment were analyzed using the NIH's *Imag*eJ software. Stomatal aperture ratio was calculated as stomata width/stomata length.

### RNA Extraction and Quantitative RT-PCR Analysis

Total RNA extraction of tomato leaves was produced using TRIzol reagent (Invitrogen, Carlsbad, CA, United States) following the manufacturer's protocol. Then, RNA was precipitated by adding one volume of 6 M LiCl for 3 h. Thereafter the pellet was washed using 1 volume of 3 M LiCl and resuspended in RNase-free water. So as to remove genomic DNA, 2 U/μL RNA of TURBO DNase kit (Ambion, Austin, TX, United States) was utilized.

PrimeScript RT reagent kit (Perfect Real Time, Takara Bio Inc., Otsu, Shiga, Japan) was used to obtain the cDNA from one microgram of total RNA, according to the manufacturer's protocol. Quantitative real-time PCRs were performed as previously described (Campos et al., 2014), in 10 μL reaction volume on 96-wells plates using SYBR® Green PCR Master Mix (Applied Biosystems). Actin gene was used as the endogenous reference gene in all the experiments. Three technical replicates were performed in all the assays. The PCR primers are listed in Table S1.

### HS-SPME Extraction and GC-MS Analysis of Volatile Compounds

For the analysis of the volatile compounds, 100 mg of fully homogenized, frozen tomato leaf tissue were introduced into a 10 mL glass vial, adding subsequently 1 mL of a saturated CaCl_2_ solution and 100 μL of 750 mM EDTA at a pH of 7.5. The vial was air-tight sealed and sonicated for 5 min and volatile compounds extraction was performed by Head Space Solid-Phase Microextraction (HS-SPME) (López-Gresa et al., 2017). The samples were incubated for 10 min at 50°C and the extraction was performed at the same temperature for 20 min with a PDMS/DVB fiber (Supelco, Bellefonte, PA, USA). The desorption of the adhered compounds was carried out for 1 min at 250°C in splitless mode. Solid phase microextraction was performed using a CombiPAL autosampler (CTC Analytics, Zwingen, Switzerland).

VOCs were analyzed using an Agilent 6890N (Santa Clara, CA, USA) gas chromatograph coupled to an Agilent 5975B Inert XL electronic impact (EI) mass detector with an ionization energy of 70 eV and a source temperature of 230°C. Chromatography was carried out on a DB-5 ms fused silica capillary column (60 m long, 0.25 mm i.d., 1 μm film thickness). The temperature conditions established in the oven were 40°C for 2 min, a ramp from 5°C/min to 250°C and 5 min at a constant temperature of 250°C. The carrier gas was helium at a constant flow of 1.2 mL/min. Data acquisition was performed at 6 scans per second in an *m/z* range of 35–250. Chromatograms and mass spectra were acquired and processed using the Enhanced ChemStation software (Agilent).

Identification of the compounds was performed by using commercial compounds that served as standards. The compounds used as reference or standard were purchased in Sigma-Aldrich (Madrid, Spain) or chemically synthesized by the Metabolomics service of the IBMCP as is the case of (*Z*)-3-hexenol esters (González-Mas et al., 2011). Quantification of HA, HB, HP, and HiB was performed by standard curves using such pure compounds.

### HB Treatments in Different Species

*Nicotiana benthamiana, Arabidopsis thaliana, Medicago sativa, Zea mays*, and *Citrus x jambhiri* plants were treated with (*Z*)-3-hexenyl butyrate (HB). For this purpose, plants were treated by spraying them at a concentration of 2 mM containing 0.05% Silwet L-77. Tomato plants were also sprayed as a positive control. Samples were taken before the treatment, and 24 and 48 h post-treatment in order to determine stomatal aperture ratio (as previously described).

### *In vitro* Antimicrobial Activity Assays

*Pseudomonas syringae* pv. *tomato* DC3000 Δ*avrPto* bacterial strains were grown in LB agar medium during 48 h and then transferred into 15 mL of King's B liquid medium, as previously described. After 24 h, 1 mL of the bacterial culture was mixed with 14 mL of King's B agar and poured into Petri dishes. Once solidified, 5 mm-diameter Whatman paper disks (GB005 Blotting Paper, Schleicher & Schuell) were placed on top of the agar and then a volume of 10 μL of each compound [(*Z*)-3-hexenyl acetate (HA), (*Z*)-3-hexenyl propionate (HP), (*Z*)-3-hexenyl butyrate (HB), (*Z*)-3-hexenyl isobutyrate (HiB), (*Z*)-3-hexenal, and (*E*)-2-hexenal] was applied into the disks. Methanol was used as negative control and the antibiotic tetracycline was chosen for the positive one at two different concentrations (50 and 100%).

After incubation during 48 h at 28°C, the inhibition zone was measured using a slide gauge.

### Statistical Analysis

Statistical analyses of two or more variables were performed by using Student's *t*-test or analysis of variance (multifactor ANOVA), respectively (Statgraphics Centurion XVI).

For untargeted analysis of the volatile profile, GC-MS data were processed with the MetAlign software (Wageningen, The Netherlands) for alignment of chromatograms and quantitation of each of the MS features. The resulting dataset was submitted to a Partial Least Square (PLS) study by means of the SIMCA-P software v. 11.0 (Umetrics, Umeå, Sweden) using unit variance (UV) scaling.

### Accession Numbers

Sequence data from this article can be found in the GenBank/EMBL and Solgenomics data libraries under accession numbers *PR1* (X68738), *P23* (X70787), *AAT1* (KM975321), *JAZ7* (Solyc11g011030), *JAZ9* (Solyc08g036640), *P5CS* (Solyc06g019170), *RAB18* (Solyc02g084850), and *Actin* (AB695290; Solyc04g011500).

## Results

### Exogenous Treatments With GLV Esters Close Stomata and Induce PR Gene Expression

**Figure 1:**
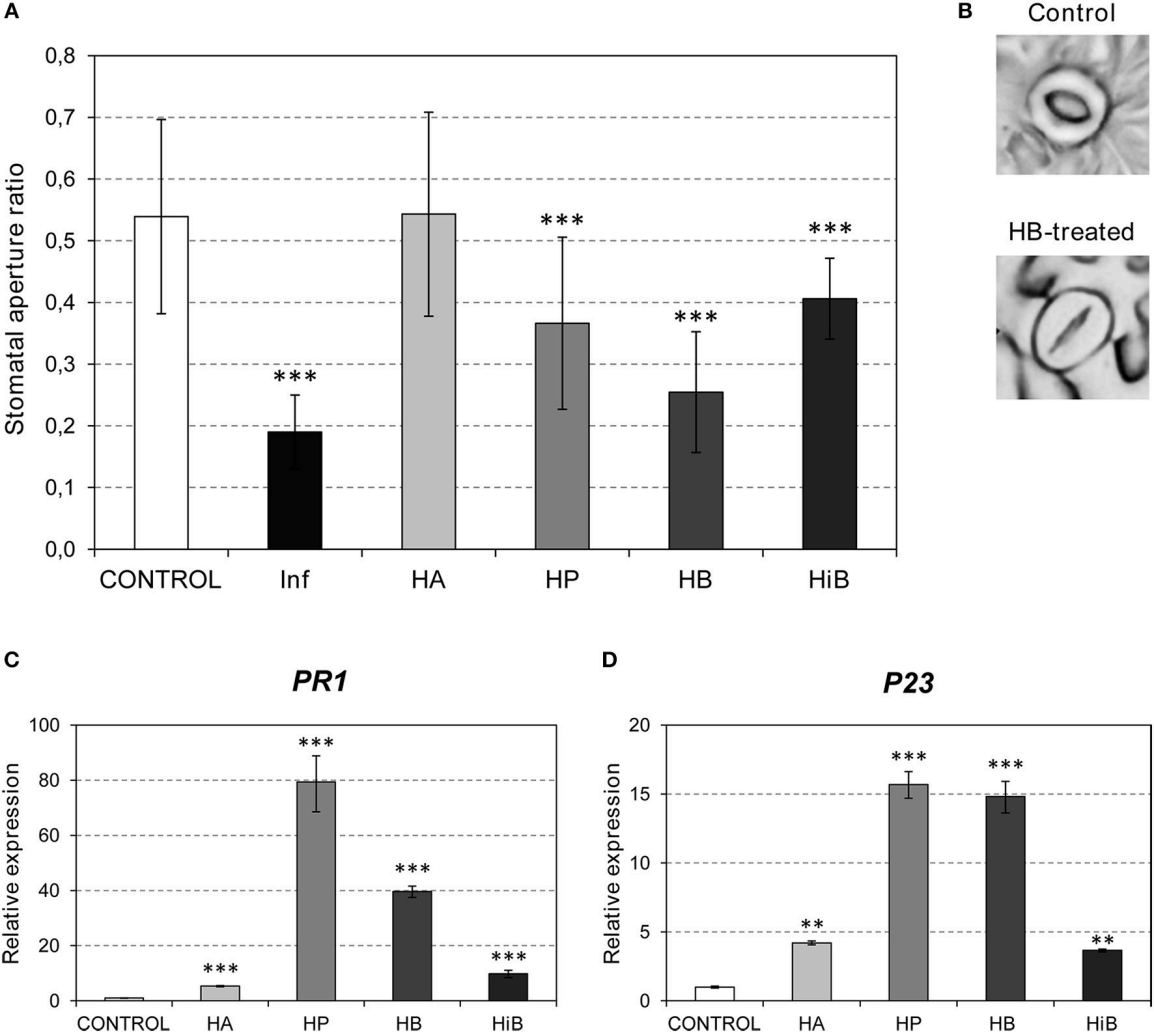
Effects of (*Z*)-3-hexenol acetic (HA), propionic (HP), isobutyric (HiB) or butyric (HB) esters on stomatal aperture and PR gene expression of Flora-Dade tomato leaves. Samples were collected 24 h after treatments. **(A)** Stomatal aperture ratio mean values ± SD of three biological replicates from a representative experiment. Asterisks (^***^) indicate statistically significance differences from control (*p* < 0.0010) and treated or *P. syringae* pv. *tomato* DC3000 infected (Inf) plants. **(B)** Representative images of stomata of control and HB-treated plants. **(C)** and **(D)** Expression of the tomato *PR1* and *P23* genes in plants after treatments with the different esters. Values were normalized to Actin. Expression levels are represented as mean ± SD of three biological replicates of one representative experiment. Double asterisk (^**^) and triple asterisk (^***^) indicate statistically significance differences between control and treated plants with *p* < 0.01 and *p* < 0.001, respectively.

Bacterial pathogens and VOCs invade the plant through stomata (Matsui, 2016; Panchal and Melotto, 2017). We decided to test the defensive role of GLV esters by studying the effect of exogenous treatments with these volatiles on stomatal closure. Treatments with HA, HP, HB, and HiB were performed on tomato cv. Flora-Dade plants (see Materials and Methods) and the stomatal aperture ratios were measured 24 h after treatment. As observed in Figures 1A,B, treatment with HB produced significant stomatal closure, reaching aperture ratios comparable to those observed in bacterial infected leaves (Lisón et al., 2017). A similar effect was detected when treatments were performed with HP or HiB, although lower effects were observed when compared with those observed for HB. However, HA had no clear effect on stomatal closure, indicating that the effect on stomatal closure is ester-specific. Interestingly, HA was the only ester displaying an antibacterial defensive role, as shown in Figure S1.

Several signal molecules have been implicated in plant immunity. SA has been mainly associated with resistance against biotrophic and hemibiotrophic microbes such as *Pst*, while the JA pathway activates resistance against necrotrophs in Arabidopsis (Robert-Seilaniantz et al., 2011). To test the VOC-mediated activation of these pathways, we studied the induction of several marker genes. *PR1* and *P23* (Rodrigo et al., 1993; Tornero et al., 1993) were used as markers for the plant response to biotrophic pathogens. To study the involvement of the JA pathway, *JAZ* genes (Ishiga et al., 2013) and *TCI21* (Lisón et al., 2006) were used. Since ABA has been classically associated with stomatal closure, the induction of *RAB18* and *P5CS1* was also analyzed (González-Guzmán et al., 2014). As shown in Figures 1C,D, induction of *PR1* and *P23* was observed in the plants treated with any of the VOCs, with HP provoking the highest induction of both PR genes. Interestingly, the markers associated with the ABA pathway were not activated by treatments with any of the GLV esters (Figure S2). In contrast, both *JAZ7* and *TCI21* were repressed by HB, but clearly induced in the HiB-treated plants. These results suggest that the stomatal closure caused by HP, HB, and HiB is not accompanied by activation of the ABA pathway, and that the JA pathway is repressed by HB and activated by HiB. Nevertheless, all of the GLV esters appeared to induce the SA pathway (Figures 1C,D).

### Treatment With (*Z*)-3-hexenyl-butyrate Induces Resistance to *Pst* in Tomato in a SA-Independent Manner

**Figure 2:**
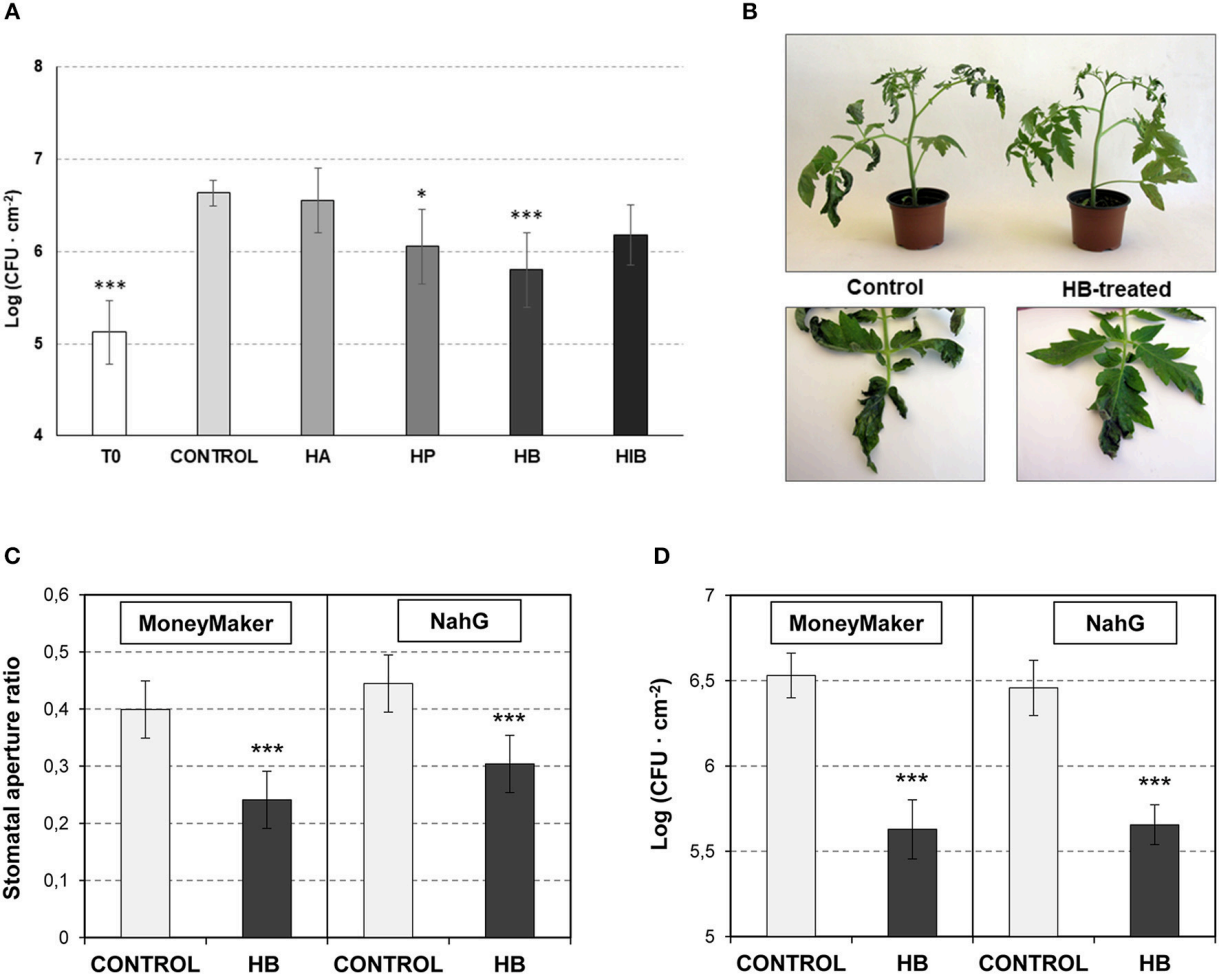
Effect of GLV ester treatments on resistance to *Pst* and involvement of SA on stomatal closure defense. **(A)** Growth of *Pst* on leaves of control and treated-plants with **(***Z*)-3-hexenyl acetate (HA), (Z)-3-hexenyl propionate (HP), (Z)-3-hexenyl butyrate (HiB) and (*Z*)-3-hexenyl-butyrate (HB). Plants were treated or not (Control) for 24 h with HA, HP, HiB or HB, and then infected with *Pst*. Samples were collected at 24 h post-infection and CFU were measured. Each bar represents the mean ± SD of three biological replicates of one representative experiment. **(B)** Representative symptomatology of control and HB-treated tomato plants at 3 days post inoculation with *Pst*. **(C)** and **(D)** Effects of treatments with (*Z*)-3-hexenyl-butyrate (HB) on the stomatal aperture **(C)** and on the resistance against *Pst* **(D)** of Moneymaker and *NahG* leaves. Plants were treated (HB) or not (CONTROL) for 24 h with HB, and then infected with *Pst*. Samples were collected 24 h after treatments for the stomatal aperture analysis. The growth of *Pst* was evaluated 1 day upon infection, by measuring the colony forming units (CFU). Asterisk (^*^) and triple asterisk (^***^) indicate significance differences with respect to control plants with *p* < 0.05 and *p* < 0.001, respectively.

Based on the observed results, we decided to test whether the observed VOC-mediated *PR* induction and stomatal closure increases resistance to bacterial infection. To that purpose, tomato plants were pre-treated with VOCs for 24 h and subsequently infected with *Pst*. Only treatments with HP and HB significantly reduced bacterial growth (Figure 2A). These results appear to indicate that the stomatal closure accounts very importantly for the tomato resistance to bacteria (Figure 1A). Although HA displayed antibacterial properties (Figure S1) and HiB induced PR1 and P23 (Figures 1C,D), any of these two VOCs could enhance the tomato resistance to *Pst*. In this sense, the highest resistance observed in the HB-treated plants, with a clear effect also in the observed symptomatology (Figure 2B), correlated with the highest efficacy of this VOC on stomatal closure. Our results suggest that HB, and to a lesser extend HP, effectively lead to resistance, confirming an indirect defensive role of both GLV esters.

Beyond its role as a signaling molecule for plant defense responses to bacterial pathogens, SA is a positive regulator of stomatal defense (Panchal and Melotto, 2017). To test if the observed HB-induced stomatal closure is SA-dependent, we studied its effect in *NahG* tomato plants, which are impaired in SA accumulation (Brading et al., 2000).

*NahG* plants and their corresponding Moneymaker parental control were pre-treated with HB for 24 h and subsequently infected with *Pst*. Stomatal aperture ratio and bacterial growth were measured at 24 h post-inoculation. HB treatment resulted in a significant stomatal closure and reduced bacterial growth in *NahG* (Figures 2C,D), indicating that the effect of HB is SA-independent.

### Antisense *as-AAT1* Plants Are More Susceptible to *Pst* Displaying a Reduction of *AAT1* but No Difference in *PR1* Upon Infection With *Pst*

To better understand the relationship between GLVs and pathogen defense, we examined plants that are impaired in their ability to synthesize GLVs. The last reaction for the biosynthesis of GLVs is carried out by an alcohol acyltransferase (AAT). In tomato, there are five AAT-encoding genes but only one, SlAAT1, accounts for almost all of the expression in fruit, and antisense *SlAAT1* fruits produce almost no detectable alcohol esters (Goulet et al., 2015).

**Figure 3:**
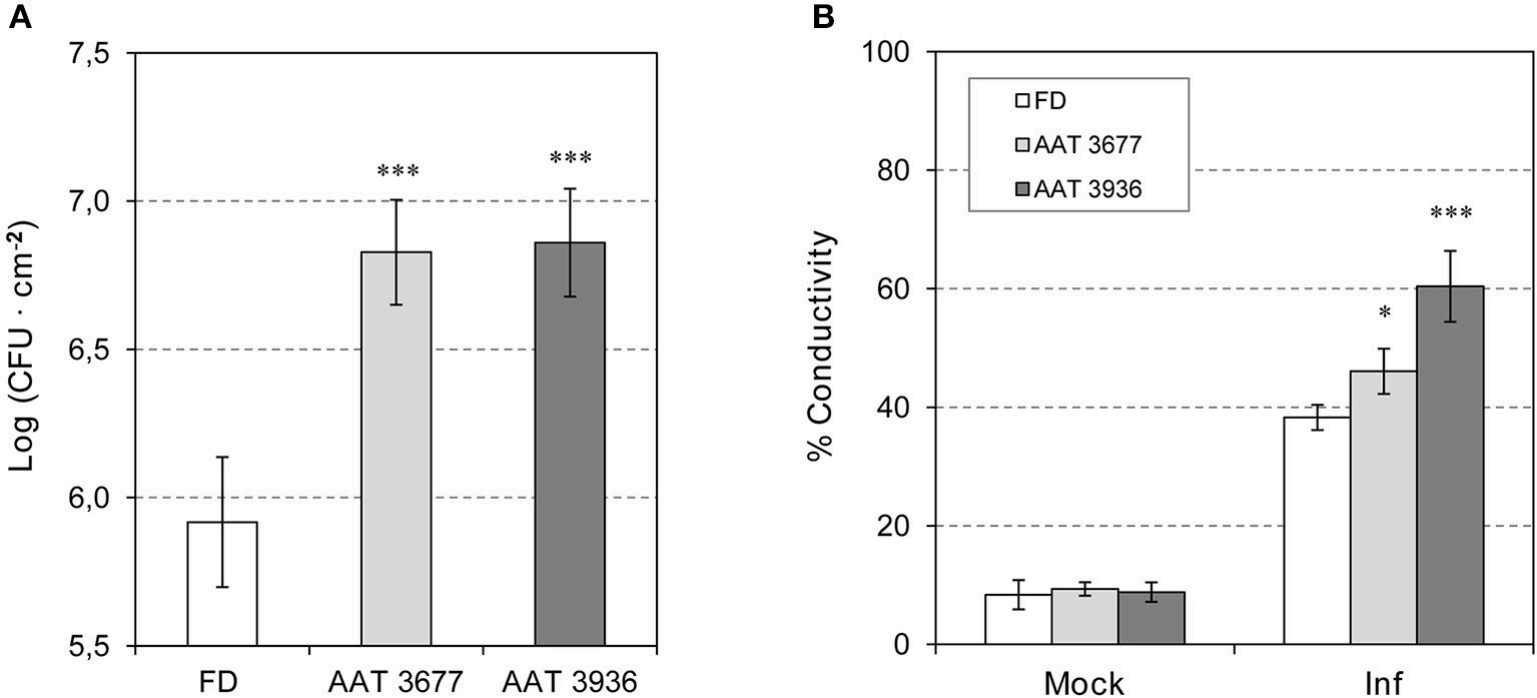
Susceptibility of antisense *as-AAT1* plants to *Pst*. **(A)** Growth of *Pst* on leaves of Flora-Dade (FD) and antisense AAT transgenic plants (AAT). Leaf colony-forming units (CFU) of bacteria were measured in leaves from wild-type (FD) and transgenic lines AAT 3677 and AAT 3936. Bacterial growth was measured 24 h after inoculation. Results correspond to means ± SE of five independent plants from a representative experiment. Data are derived from three independent experiments. **(B)** Cell death of FD and antisense AAT transgenic tomato plants (AAT) infected with *Pst*. Cell death of tomato 24 h after challenge with virulent *Pst* in wild-type (FD) plants and transgenic lines AAT 3677 and AAT 3936. Cell death was measured in the form of percent ion leakage in plants treated with a mock challenge or challenged with virulent bacteria. Data are derived from three independent experiments. Asterisks (^*^) and (^***^) indicate significance with a *p* < 0.05 and *p* < 0.001 with respect to WT plants, respectively.

To determine whether *SlAAT1* has an active role in plant defense, two independent *as-AAT1* transgenic lines (AAT 3677 and AAT 3936) (Goulet et al., 2015) were infected with *Pst*, and the bacterial populations were measured and compared to the corresponding Flora-Dade control plants. As shown in Figure 3A, *as-AAT1* plants had significantly more bacterial growth at 24 hpi than did the control plants. Electrolyte leakage, which is a hallmark of cell death, was also significantly increased in both *as-AAT1* transgenic lines (Figure 3B). Our results indicate that reduced expression of *SlAAT1* strongly compromises the defense response against *Pst*.

To characterize the response of *as-AAT1* tomato plants to *Pst* infection, the expression of different genes in *as-AAT1* lines and Flora-Dade plants either mock-inoculated or infected with *Pst* at 24 and 48 hpi was analyzed by qRT-PCR (Figure S3). As previously described in cv. Rio Grande tomato plants (López-Gresa et al., 2017), there was an increase in *SlAAT1* transcript in *Pst*-infected Flora-Dade leaves when compared to the mock-inoculated plants, at both 24 and 48 hpi. In contrast, a clear reduction in *SlAAT1* was observed in *as-AAT1* leaves relative to the Flora-Dade control plants in all conditions (Figure S3A).

We also studied the induction of *PR1*, observing that activation of this gene only occurs in the presence of the bacteria, being stronger at 48 hpi (Figure S3B). No significant differences in the induction of *PR1* between Flora-Dade and the *as-AAT1* lines were observed after *Pst* infection. These results suggest that the enhanced susceptibility observed in *as-AAT1* transgenic plants correlated with lower expression of *AAT1*, but not with the pathogenesis marker *PR1*.

### Antisense *as-AAT1* Plants Emit Less GLV Esters and Display an Impaired Stomatal Closure Upon *Pst* Infection

In order to correlate the observed phenotype with the differential emission of GLV esters, volatiles produced by *as-AAT1* and Flora-Dade leaves either mock-inoculated or infected with *Pst* at 24 and 48 hpi were analyzed by GC-MS.

To manage the large amount of mass data, a multivariate data analysis was performed. In particular, a partial least square analysis (PLS) was carried out, defining compound abundance as the X variable, and harvest time (24 and 48 hpi) and genotype (FD; AAT 3677 and AAT 3936) as stepwise Y variables. The PLS analysis (Figure S4) showed that the first component (PC1) explained changes in the chemical composition along the time course of the infection (harvest time) while the metabolic alterations due to the genotype were clearly characterized by the second component (PC2). Both *as-AAT1* infected transgenic samples showed an evident variation in their metabolic content compared to FD infected plants. The analysis of loading scatter plot identified the GLV esters as the main compounds contributing to the separation of the samples.

Levels of (*Z*)-3-hexenyl-acetate, -propionate, -isobutyrate, and -butyrate were specifically quantified. As shown in Figure S5, these four esters of (*Z*)-3-hexenol were differentially emitted by Flora-Dade plants upon infection with *Pst*, thus extending the previously described emission observed in Rio Grande plants (López-Gresa et al., 2017) to other tomato cultivars. It is worth noting that (*Z*)-3-hexenyl-butyrate displayed the highest levels of emission (around 50 ppm) in Flora-Dade leaves 24 h after the *Pst* inoculation, reaching values even higher than those used for the HB treatments (around 1 ppm). As expected, both *as-AAT1* lines infected with *Pst* differentially emitted less GLV esters when compared with the corresponding Flora-Dade infected plants, displaying levels of emission similar to the mock-inoculated *as-AAT1* leaves. Our results suggest that these GLVs may play a defensive role in tomato plants against bacterial infection.

**Figure 4:**
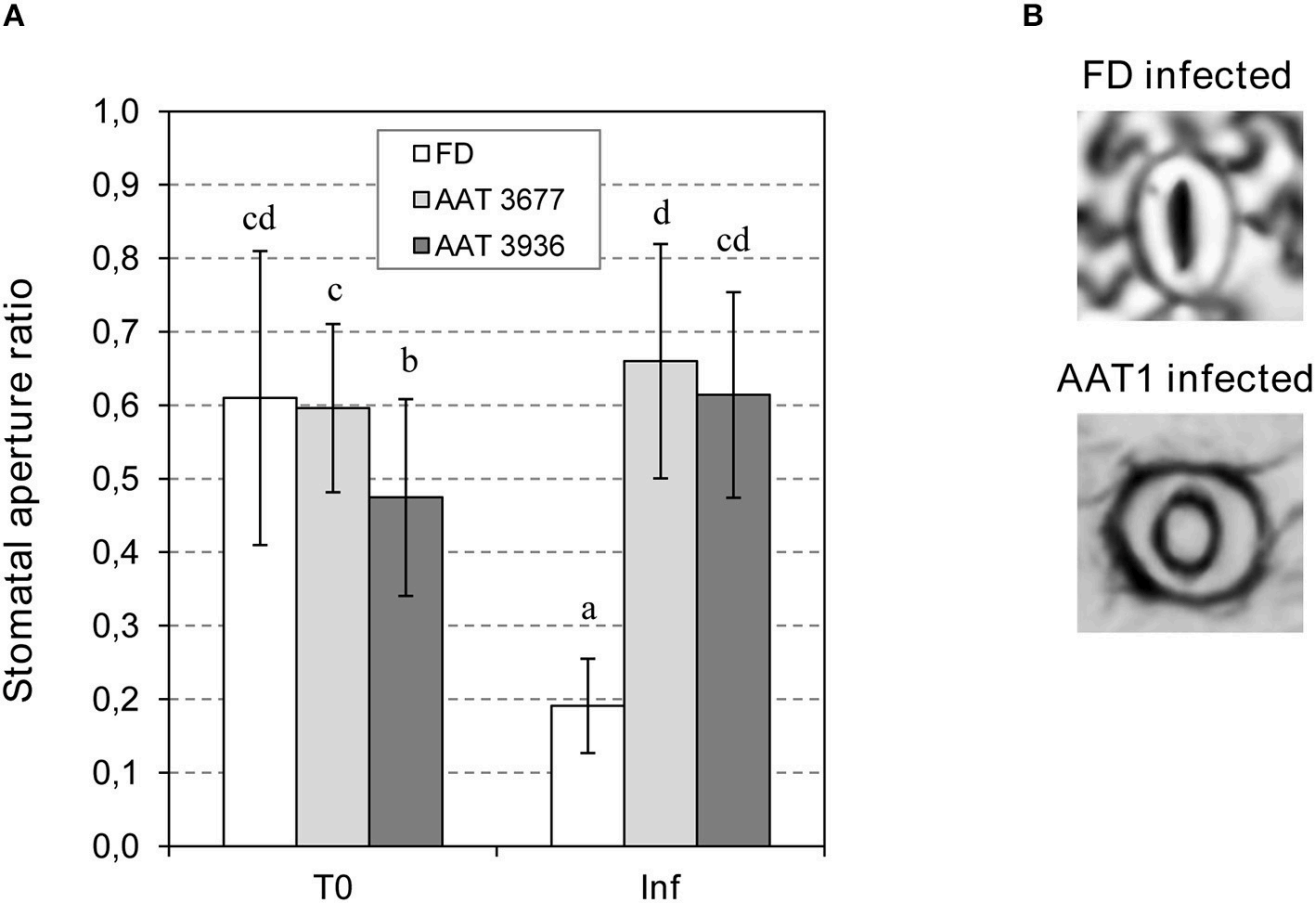
Stomatal opening in Flora-Dade (FD) and AAT antisense transgenic plants infected with *Pst*. **(A)** Ratio of stomatal opening (r/R) in leaves of AAT1 lines (AAT3677 and AAT3936) and FD leaves 24 h after inoculation with *Pst*. An ANOVA test was performed and different letters indicate the statistical significances with a *p*-value < 0.05 **(B)** Representative images of stomata of FD and AAT infected plants.

To study the possible effect of GLV esters on bacterial-mediated stomatal closure, we measured the stomatal aperture ratio in *as-AAT1* and Flora-Dade leaves, both before and 24 h after bacterial infection. As shown in Figure 4, before infection, both *as-AAT1* lines had similar ratios of stomatal aperture to Flora-Dade. However, the stomata of Flora-Dade leaves displayed a reduction in the aperture ratio after *Pst* infection. Interestingly, stomatal closure did not occur in *as-AAT1* infected leaves, exhibiting stomatal aperture ratios significantly higher than those observed in the Flora-Dade infected leaves. Our results are consistent with a model in which the increased susceptibility in *as-AAT1* against *Pst* is caused by the reduced ability of these plants to close stomata upon infection due to the lack of the GLV esters emission in these plants. These results uncover a role of GLV esters, and particularly, of HB in stomatal defense.

### The GLV Esters Defense Role Is Mainly Due to Their Stomata Closure Effect

**Figure 5:**
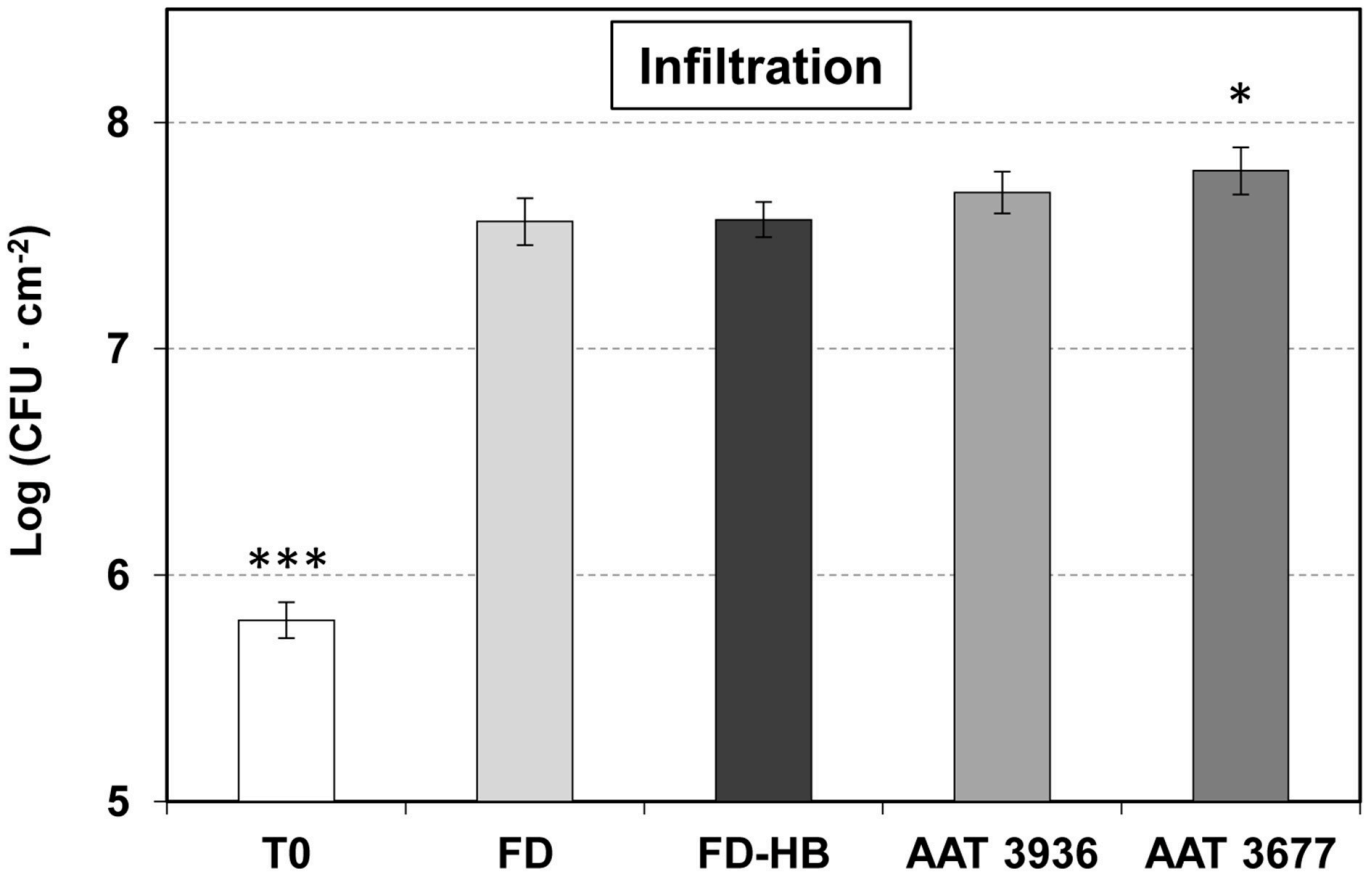
Growth of *Pst* on leaves of non-treated and HB-treated Flora Dade plants, and antisense AAT transgenic tomato plants after infiltration. Leaf colony-forming units (CFU) of bacteria were measured at 24 h after inoculation with a syringe in Flora Dade non-treated (FD) or HB-treated (FD-HB) plants and antinsense transgenic lines AAT 3677 and AAT 3936. Results correspond to means ± SE of six independent plants from a representative experiment. A *t*-test analysis was performed with the data coming from three independent experiments. Asterisks (^*^) and (^***^) indicate significance with a *p*-value < 0.05 and *p*-value < 0.001, respectively, with respect to control plants.

To test if the observed GLV esters defense role was only due to a reduction in stomatal closure, or other factors could be intervening, non-treated and HB-treated tomato Flora-Dade plants were infected by infiltration with a syringe (Figure 5). In this case, the bacteria bypass the stomatal defense and directly enter the apoplast. As expected, forced infiltration resulted in higher levels of bacterial growth, when compared to the plants infected by immersion (Figure 3). As Figure 5 shows, no statistical differences in the bacterial content were observed after HB treatments, thus indicating that the HB-induced resistance mainly depends on the stomata closure effect.

To better confirm this idea, *as-AAT1* plants were also infiltrated with *Pst* (Figure 5), observing a clear reduction in the hypersusceptibility, that was previously detected by immersion (Figure 3). Consistently a similar induction of *PR1* was observed in both Flora-Dade and *as-AAT1* plants infected with the bacteria (Figure S3B), thus suggesting that the phenotype is not due to reduced activation of plant defense, but to a reduced ability to close stomata in the *as-AAT1* plants.

All these results confirm the role of Green Leaf Volatile esters in tomato stomatal defense against *Pst*.

### Rio Grande Tomato Plants Displaying ETI Exhibit a Slightly Higher Stomatal Closure

Since Rio Grande ETI-displaying tomato plants differentially emit GLV esters at 1 h, 4 h (Figure S6), and 24 h post-inoculation (López-Gresa et al., 2017), and treatments with some of them produce stomatal closure (Figure 1), we decided to study the stomatal behavior in plants displaying ETI, compared with those infected with the virulent bacteria. To that purpose, Rio Grande tomato plants carrying the *Pto* gene were infected with an avirulent or a virulent strain of *Pst*, carrying the *AvrPto* gene or a deleted version of it, respectively (see Materials and Methods). The stomatal closure ratios were measured at 1, 4, and 24 h post-inoculation (Figure S7). In correlation with the differentially emitted levels of GLV esters, tomato plants displaying ETI appeared to exhibit a slightly higher stomatal closure at the early analyzed times.

### HB Treatments Produce a Maximum Stomatal Closure at 24 h in Tomato Plants and It Is Also Effective in Several Plant Species

**Figure 6:**
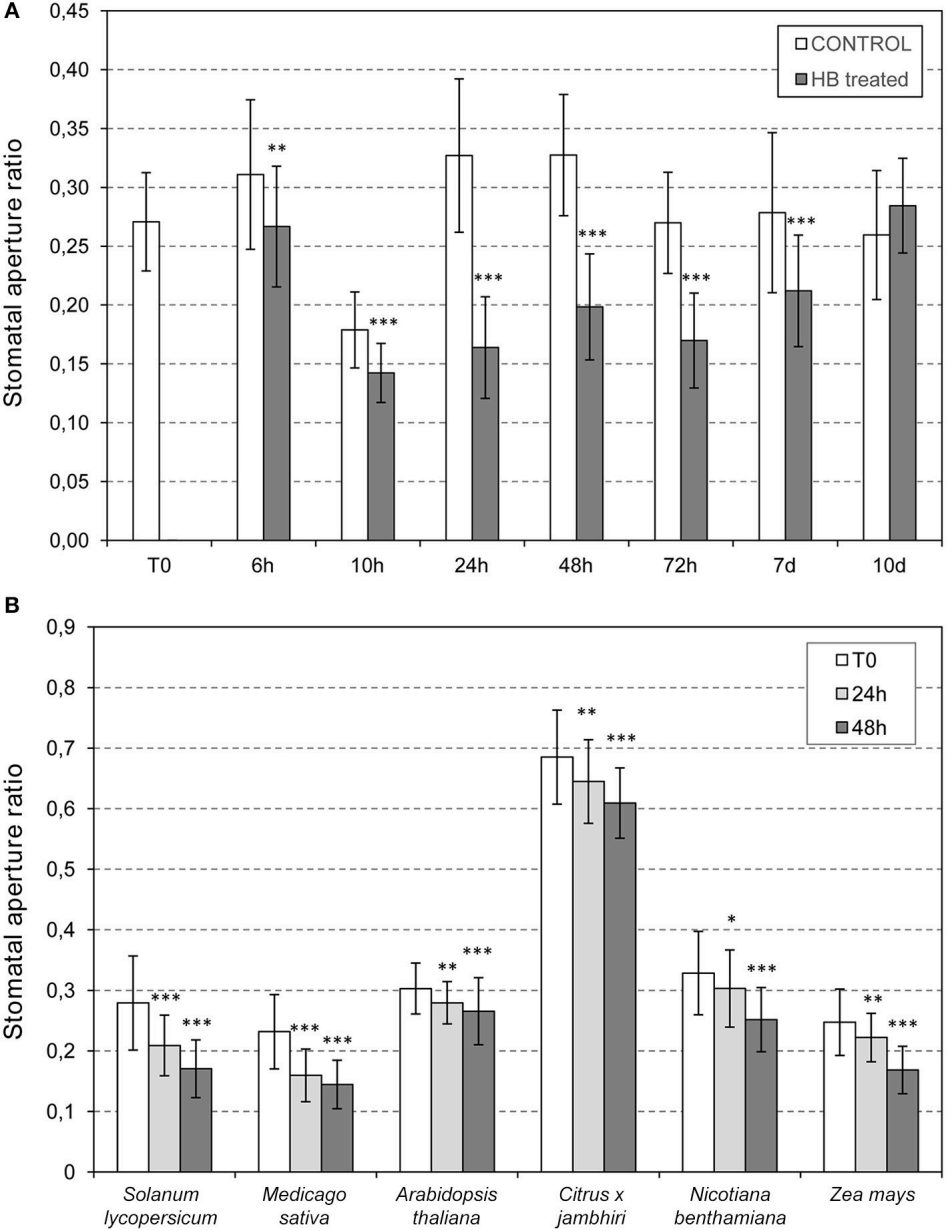
Stomatal opening in plants treated with (*Z*)-3-hexenyl-butyrate (HB). **(A)** Time course analysis of the (HB) effect on tomato stomata closure. Stomata ratios were analyzed in non-treated (control) and HB-treated (HB) tomato leaves at 0 (T0), 6, 10, 24, 48, 72 h post-treatment (h), and at 7 and 10 days post-treatment (d). **(B)** Effectivity of HB treatments in different species. Samples were collected 24 or 48 h after treatments. Asterisk (^*^), double asterisks (^**^) and triple asterisks (^***^) indicate significant differences between control and HB-treated plants with *p* < 0.05, *p* < 0.01 and *p* < 0.001, respectively.

To study the most effective time point for the HB-induced stomata closure, measures of stomata ratios were analyzed in control and HB treated tomato leaves at 6, 10, 24, 48, 72 h post-treatment (hpt), and at 7 and 10 days post-treatment (dpt). As Figure 6A indicates, HB was already effective at 6 hpt, displaying a maximum stomata closure effect at 24 hpt. At this time point, the ratio in HB-treated plants decreased to half of that displayed by the control plants, thus indicating a very pronounced effect of the GLV ester in tomato plants. The resistance of the HB-treated plants was precisely studied after 24 h of treatment (Figure 2A). The durability of the effect remained till 10 dpt, at which the differences between stomata ratios of control and HB-treated plants were not significant.

Once we had demonstrated that HB is a defensive VOC associated with stomatal closure in tomato, with a maximum effect at 24 hpt, we evaluated the effect of this GLV ester in other plant species, including *Nicotiana benthamiana, Arabidopsis thaliana, Medicago sativa, Zea mays*, and *Citrus x jambhiri*. To that end, HB treatments were performed by spraying these species, including tomato plants as a positive control, and measuring stomatal aperture ratios before and 24 or 48 h after HB treatment (Figure 6B). The treatments produced significant stomatal closure in all the species tested, thus indicating that HB is a universal stomata closer, extending its uses to Solanaceae, Brassicaceae, Fabaceae, Poaceae, and Rutaceae (Lisón et al., 2017). It is worth noting that, despite being significant, the observed effect in Arabidopsis was lower than what was observed in the rest of the analyzed species.

Our results support a model in which VOC ester synthesis is stimulated by the presence of *Pst*, triggering stomatal closure that limits entry of *Pst* into leaves and limits subsequent disease symptom development.

## Discussion

Green leaf volatiles (GLVs) consist of a family of C6 compounds, including aldehydes, alcohols, and esters, which are abundantly produced across the plant kingdom. Although the defensive role of GLVs has been classically associated with plant-herbivore interaction, it has been recently described that pathogens also provoke a higher emission of these plant volatiles (Ameye et al., 2018). In particular, some GLV esters are differentially emitted by tomato plants during establishment of the ETI triggered by *Pst* (López-Gresa et al., 2017). A direct defensive role of GLVs against pathogens has been demonstrated, since they display antibacterial activities against both gram-negative and gram-positive bacteria (Nakamura and Hatanaka, 2002). In this sense, GLVs formed during the avirulent infection of beans against *Pseudomonas* were sufficient to be toxic to the pathogenic bacteria (Croft et al., 1993). In the present work, we have also observed antimicrobial properties for HA (Figure S1), although it appears to be insufficient to enhance resistance to *Pst*. Here, we describe an indirect defensive role of GLV esters in stomatal defense of plants against *Pst*.

We observed that tomato plants treated with HP or HB display a higher ratio of stomatal closure, PR gene induction and reduced symptom development following *Pst* inoculation. Moreover, *as-AAT1* tomato plants, which are impaired in the emission of these GLV esters upon bacterial infection, exhibited a lower ratio of stomatal closure and were hyper-susceptible to *Pst*. Similar results have been described with the precursors of these volatiles in Arabidopsis (Montillet et al., 2013). These authors demonstrated that treatments with LOX substrates (PUFAs), or with LOX products (fatty acid hydroperoxides; FAHs) trigger stomatal closure, and that the 9-lipoxygenase, *LOX1*, is required to trigger stomatal closure in response to bacteria in Arabidopsis. Related to this result, we observed that Arabidopsis plants treated with HB have a higher ratio of stomata closure (Figure 6B), consistent with a role for GLV esters affecting stomatal defense in Arabidopsis as well. It would be of great interest to study the possible PUFA-mediated emission of GLV esters, or the phenotype against bacteria of Arabidopsis mutants in 13-LOX gene, which is responsible for GLV ester biosynthesis.

GLVs have been reported to induce resistance against fungal pathogens. In this context, application of (*E*)-2-hexenal reduced the severity of powdery mildew in tobacco plants (Quaglia et al., 2012). Exogenous treatments with (*E*)-2-hexenal, (*Z*)-3-hexenal, or (*Z*)-3-hexenol induced expression of several defense genes and enhanced resistance against *Botrytis cinerea* in Arabidopsis (Kishimoto et al., 2008). Also, transgenic tomato plants overexpressing a tea hydroperoxide lyase (*CsiHPL1*) release more constitutive and wound-induced GLVs, including (*Z*)-3-hexenal and (*Z*)-3-hexen-1-ol, and exhibit enhanced resistance to the necrotrophic fungus *Alternaria alternata* f. sp. *lycopersici* (Xin et al., 2014). Numerous examples of the role of GLVs in plant-fungal interactions have been reviewed (Scala et al., 2013). All these results, together with the work presented here clearly demonstrate the involvement of GLVs in the plant defense response. Furthermore, some species of fungi, such as *Puccinia* are specialized to enter the leaves only through stomata and several fungal metabolites that modulate stomatal behavior have been described (Shafiei et al., 2007; Grimmer et al., 2012; Murata et al., 2015). Therefore, GLVs may also play a role in stomatal defense against fungal pathogens.

**Figure 7:**
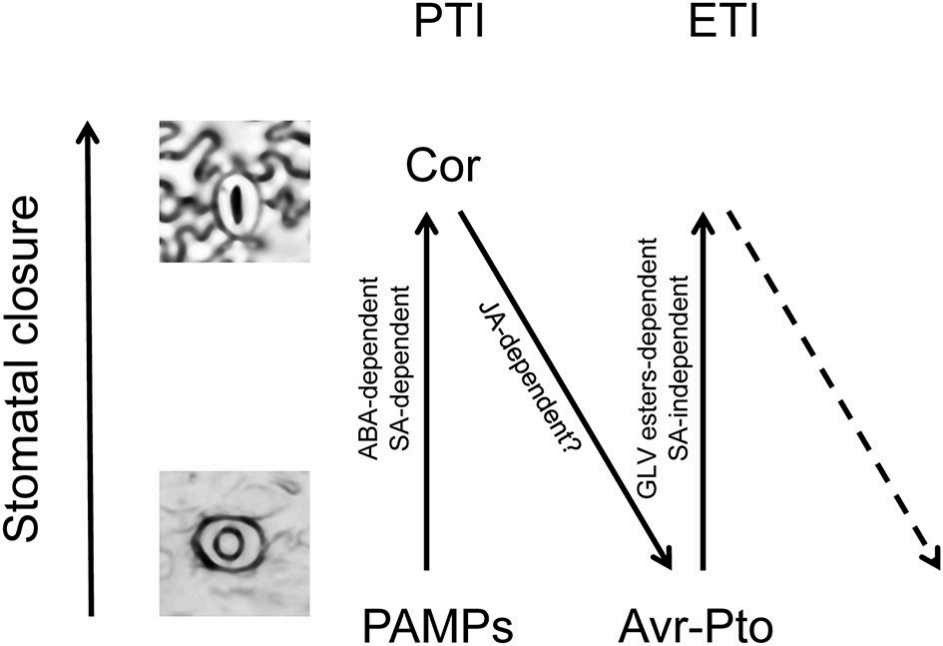
Zig-zag model for stomatal defense. Analogously to the previously proposed zig-zag model for plant immunity (Jones and Dangl, 2006), three different phases are proposed for the stomatal defense. In phase 1, upon perception of the pathogen-associated molecular patterns (PAMPs), plants close stomata in an ABA-dependent manner, contributing to the PAMP-triggered immunity (PTI). In phase 2, bacterial coronatine provokes a JA-dependent reopening of the stomata, interfering with PTI. In phase 3, the bacterial effectors are recognized by NB-LRR proteins activating effector-triggered immunity (ETI), and then the plant recloses the stomata in a GLV-dependent and SA-independent process.

Likewise, the regulation of stomatal aperture is known to be part of the plant immune response against bacteria. Two consecutive steps of stomatal movement upon infection with *Pst* in Arabidopsis (Melotto et al., 2006) and tomato (Du et al., 2014) have been described. Upon perception of the pathogen-associated molecular patterns (PAMPs), plants close stomata within 1 h in an ABA-dependent manner, thus limiting entry of the pathogen. In a second phase, bacterial coronatine provokes a JA-dependent reopening of the stomata after 3–4 h. Here we observed that, as a consequence of recognition of the bacterial effectors, a third phase may occur so that the plant recloses the stomata in a SA-independent process. Analogous to the plant immune zig-zag model (Jones and Dangl, 2006), a stomatal defense zig-zag model can be proposed (Figure 7).

Broadening the effect of GLV esters on plants, we observed that HB treatments provoke stomatal closure in several plant species, belonging to Solanaceae, Leguminosae, Brassicaceae, Citrus, and Gramineae (Figure 6B) (Lisón et al., 2017). Stomatal aperture regulates not only the entry of pathogens, but also several important processes including CO_2_ uptake for photosynthesis and loss of water by transpiration. It has been widely described that controlling stomatal closure can enhance plant resistance to drought (Cominelli et al., 2010). However, prolonged stomatal closure is not sustainable, since it limits photosynthetic assimilation and growth (Farquhar and Sharkey, 1982). We propose that application of HB may have utility in agriculture for punctuated periods to alleviate both biotic and abiotic stresses. Further studies will be necessary to better establish the HB uses.

Advances in the identification of genes and enzymes responsible for biosynthesis of volatile compounds have made possible the development of metabolic engineering, enabling improvement of different plant characteristics, including reproduction, the quality of the aroma of the fruits and defense against herbivores (Dudareva and Pichersky, 2008). Chemical communication between plants has also received much attention because of the role of the VOCs as possible inducers of defenses in host plants. In this respect, airborne signals from BTH-treated or infected plants can enhance the resistance of lima bean to a bacterial pathogen (Yi et al., 2009). Similarly, overexpressing β-ocimene synthase in tobacco plants results in higher emission of this monoterpene and induces resistance to herbivores in neighboring corn, lima beans and tomato plants (Muroi et al., 2011; Cascone et al., 2015). Moreover, antifungal volatiles released from Chinese chive help control Panama disease in banana (Zhang et al., 2013). Our results could lead to the use of metabolic engineering for improving the defense of plants against pathogens, by modifying GLV ester biosynthetic genes. The volatile nature of GLV esters would permit these transgenic plants to provide protection in non-transgenic neighboring crops.

## Author Contributions

PL and ML-G conceived and designed the study. ML-G, MO, and CP performed the experiments. CP and IR prepared the figures. ML-G and JB performed the GC-MS-based results. ML-G and PL wrote the manuscript. IR, VC, HK, and JB supervised the experiments and complemented the writing.

### Conflict of Interest Statement

The authors declare that the research was conducted in the absence of any commercial or financial relationships that could be construed as a potential conflict of interest.
