# Supplementary material for: A New Role For Green Leaf Volatile Esters in Tomato Stomatal Defense Against Pseudomonas syringe pv. tomato
Source: Front Plant Sci. 2018 Dec 18;9:1855. doi: 10.3389/fpls.2018.01855 (PMC6305539; doi:10.3389/fpls.2018.01855)
Supplement: Supplementary file 1 [file Data_Sheet_1.PDF]

Supplemental figure 1

A

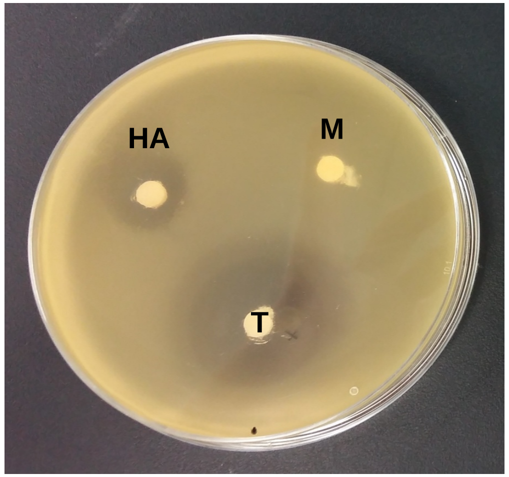

B

	% (v/v)	Mean zone of inhibition ± SD (cm)
**Tetracycline**	0.785*	2.92 ± 0.76
	0.395*	1.80 ± 0.43
**(*Z*)-3-hexenyl acetate (HA)**	100	0.87 ± 0.03
	50	0.62 ± 0.03
**(*E*)-2-hexenal**	100	7.05 ± 0.17

Figure S1. *In vitro* antimicrobial activity of different VOCs. Bacteria *Pseudomonas syringae* pv. *tomato* DC3000 was grown in King B medium. The area of inhibition was measured 48h after compounds' addition. **A,** Representative growth inhibition test of 100% (v/v) concentrated (*Z*)-3-hexenyl acetate (HA) and methanol (M), and 0.785% (w/v) tetracycline. **B,** Mean zone of inhibition ± SD for tetracycline, (*Z*)-3-hexenyl acetate and (*E*)-2-hexenal at different concentrations.

Supplemental figure 2

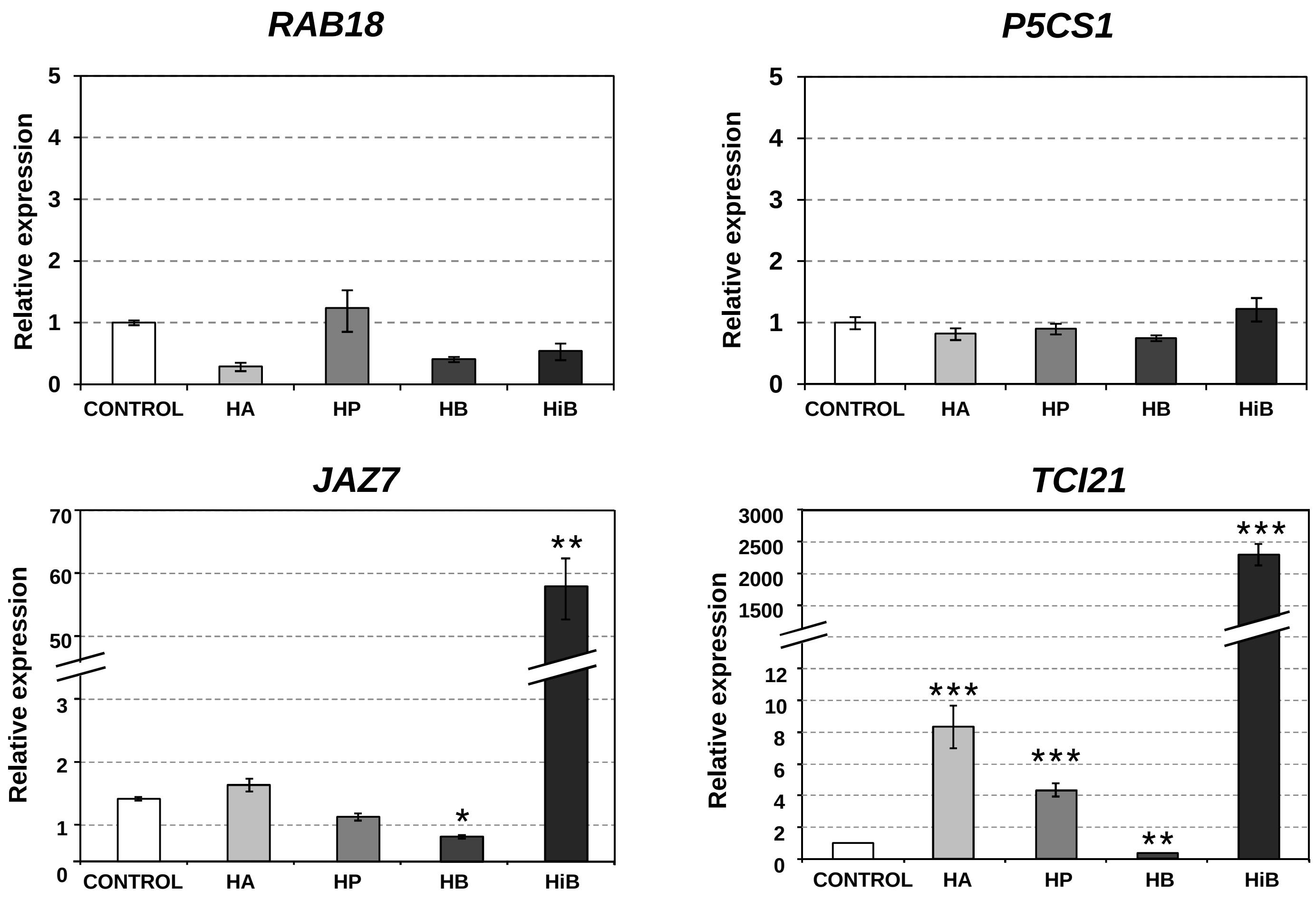

Figure S2. Expression of the tomato *JAZ7*, *TCI21*, *RAB18* and *P5CS1* genes in control and treated-plants 24 h after treatments with acetic (HA), propionic (HP), isobutyric (HiB) or butyric (HB) (*Z*)-3-hexenol esters. Transcript levels were determined by a real-time qRT-PCR analysis. Values were first normalized to the level of actin transcript. Expression is presented as mean ± SD of three biological replicates of one representative experiment. Asterisk (*), double asterisks (**) and triple asterisks (***) indicate significant differences between control and treated plant with $p < 0.05$, $p < 0.01$ and $p < 0.001$, respectively.

Supplemental figure 3

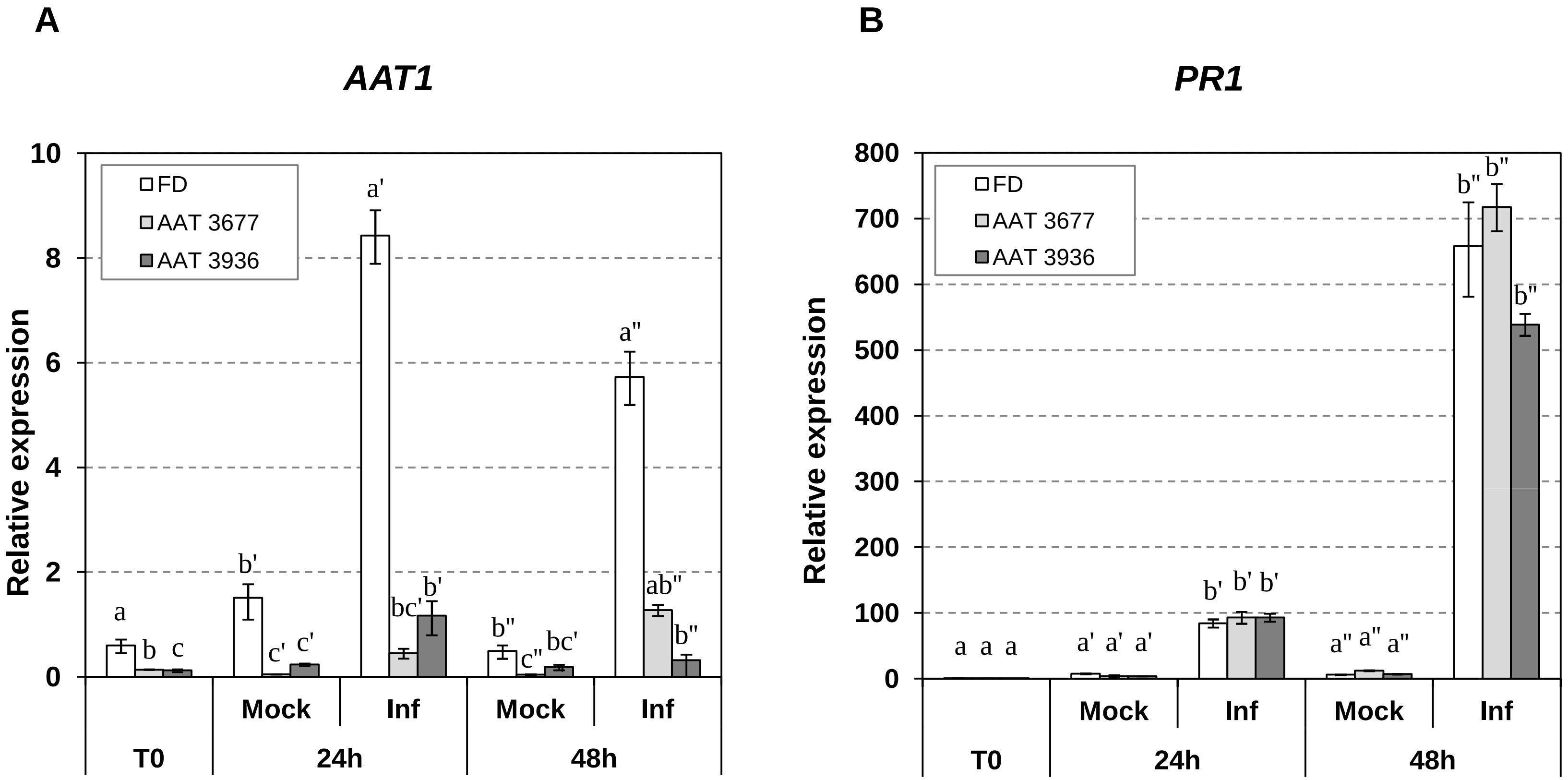

Figure S3. Expression of *AAT1* (a) and *PR1* (b) in Flora Dade (FD) and AAT antisense transgenic tomato plants infected with *Pst*. Gene expression was studied in mock-inoculated Flora-Dade plants (Mock) and upon infection with *Pst* (Inf) at 24 and 48 h post-inoculation. qRT-PCR values were normalized to Elongation Factor 1 alpha (eEF1α) level. Values are mean +/- SE of three replicates. An ANOVA test was performed and different letters indicate the statistical significances with a $p < 0.05$. ANOVA letters x, x', and x'' correspond to T0, 24 h and 48 h samples, respectively.

Supplemental figure 4

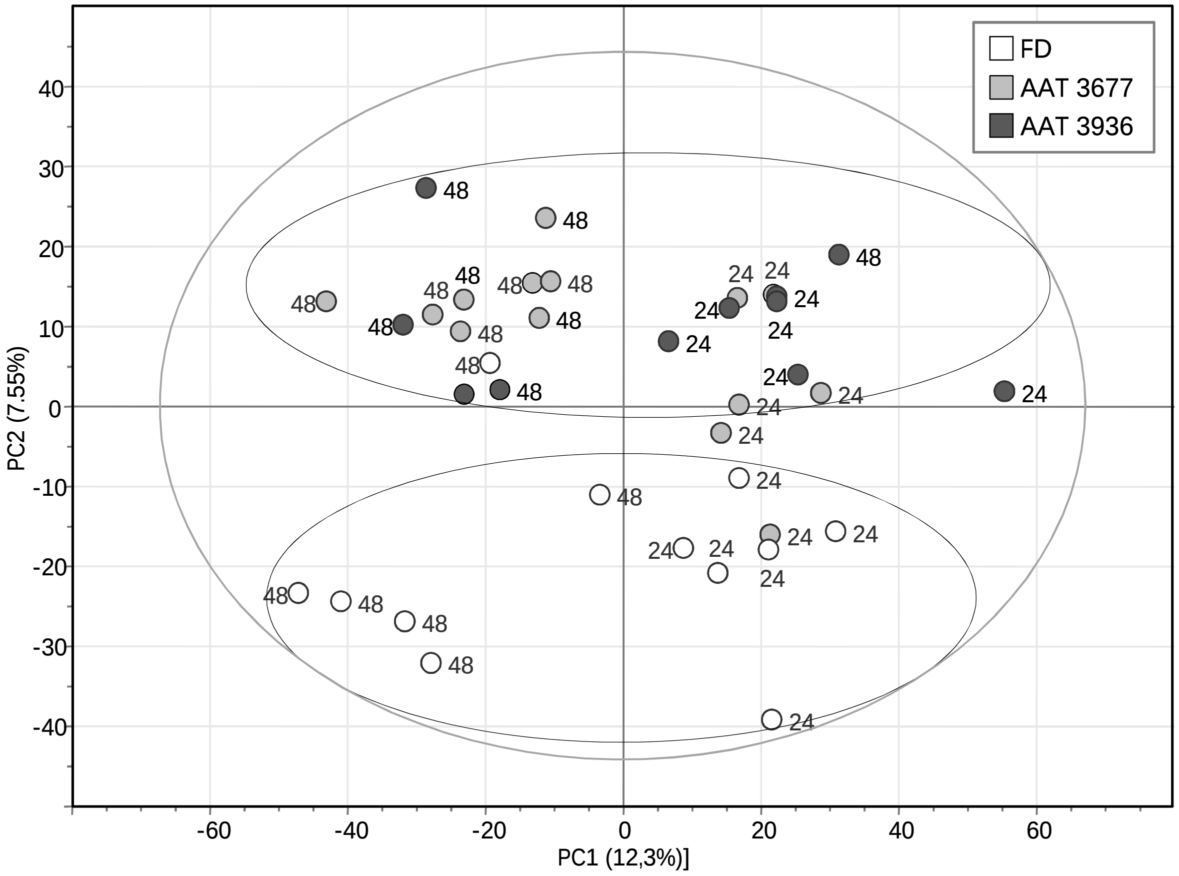

Figure S4. Score plot of PLS based on the whole array of the mass spectra in a *m/z* range from 35 to 250. Leaves of Flora-Dade (FD) and AAT transgenic plants (AAT 3677 and AAT 3936) upon infection with *Pst* at 24 (24), and 48 (48) h post-inoculation.

Supplemental figure 5

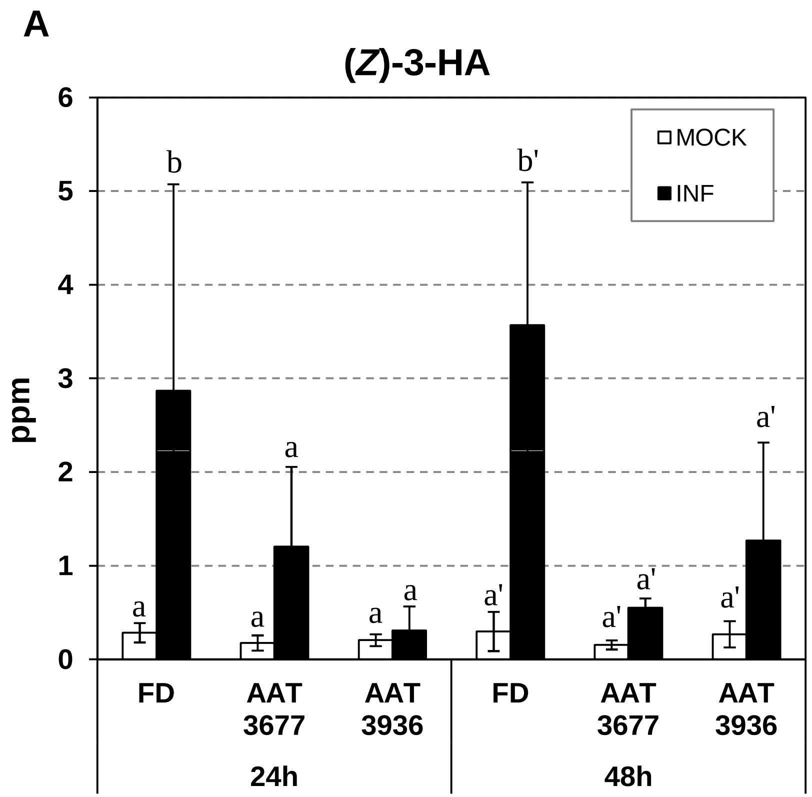

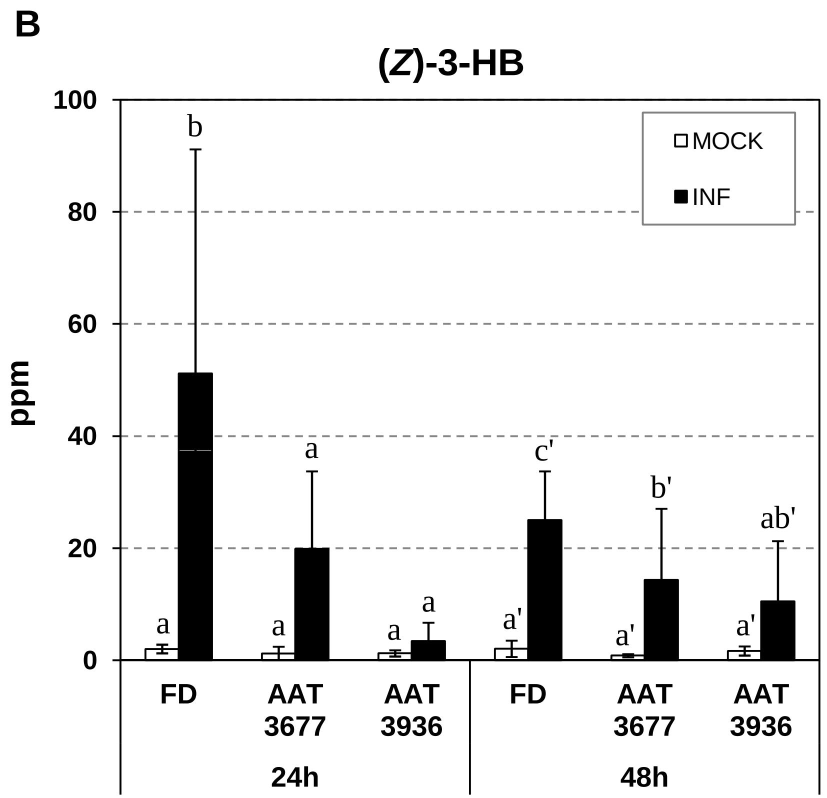

Supplemental figure 5, continued

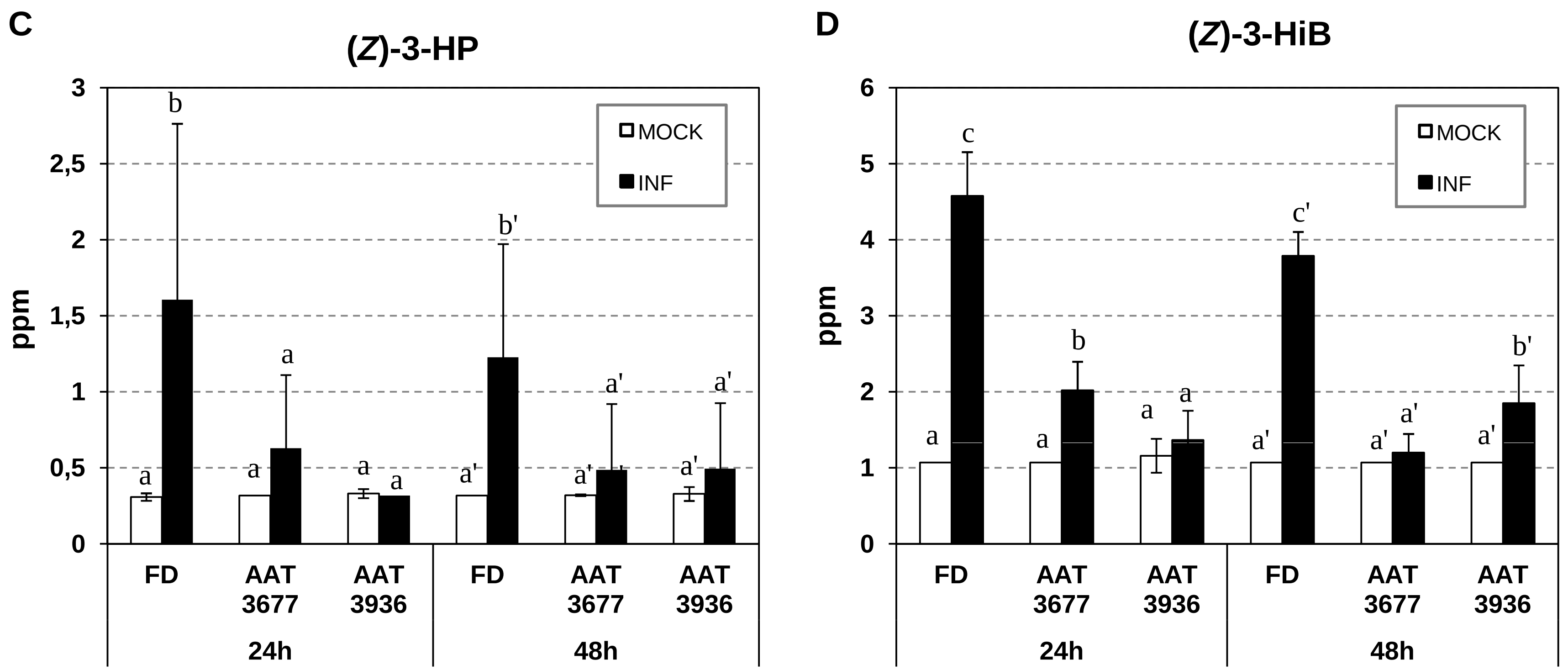

Figure S5. VOCs emission by Flora Dade (FD) and AAT antisense transgenic leaves upon infection with *Pst*. A, Emission of (*Z*)-3-hexenyl-acetate (HA) (panel **A**), (*Z*)-3-hexenyl-butyrate (HB) (panel **B**), (*Z*)-3-hexenyl-propionate (HP) (panel **C**) and (*Z*)-3-hexenyl-isobutyrate (HiB) (panel **D**) in mock-inoculated plants (MOCK) and upon infection with *Pst* (INF) at 24 and 48 h post-inoculation using Flora-Dade (FD) and AAT transgenic lines (AAT 3677 and AAT 3962). An ANOVA test was performed and different letters indicate the statistical significances with a *p*-value < 0.05

Supplemental figure 6

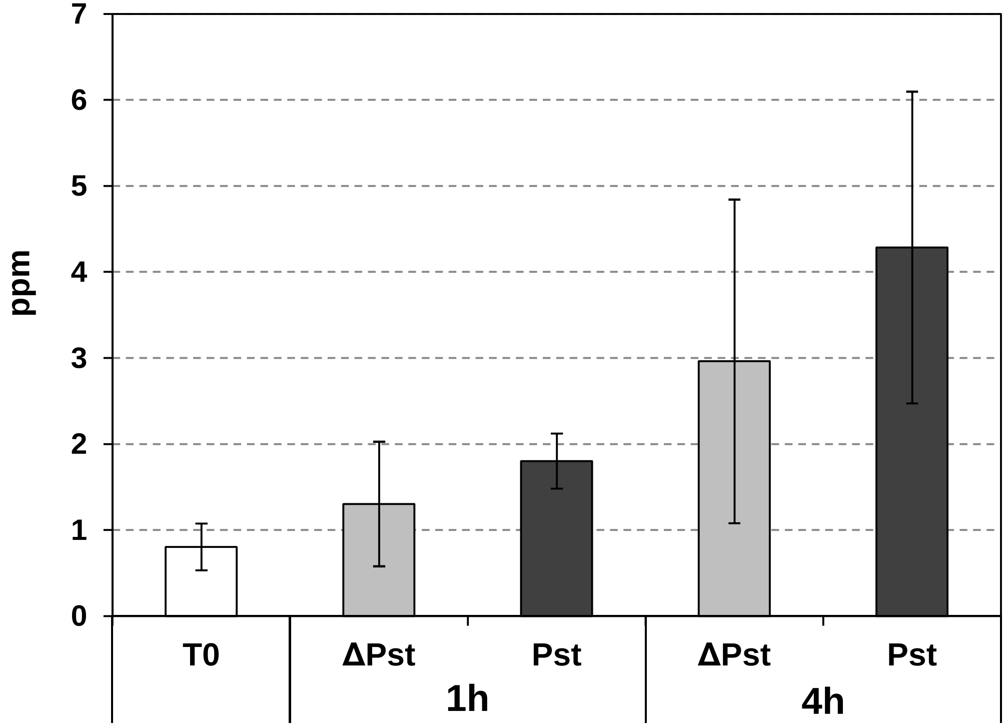

Figure S6. (*Z*)-3-hexenyl-butyrate emission by Rio Grande tomato leaves upon infection with *Pst* and *Pst* containing deletions in avrPto and avrPtoB (ΔPst). Samples were collected 1 h and 4 h after infections. Bars represent (*Z*)-3-hexenyl-butyrate emission ± SD of three biological replicates of one representative. There were no statistical differences between tomato plants infected with *Pst* and those infected with *Pst* ΔavrPto/ΔavrPtoB at any time.

Supplemental figure 7

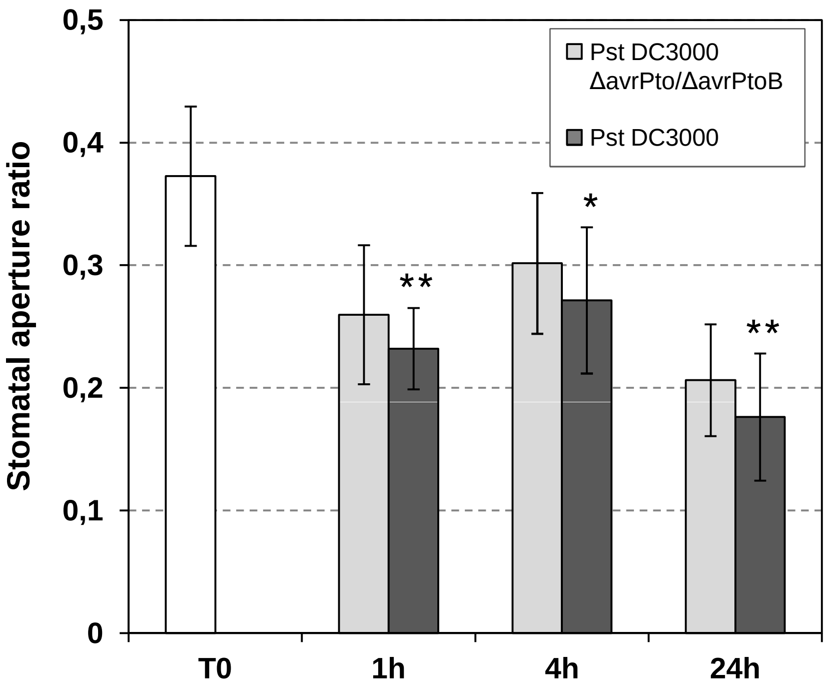

Figure S7. Stomatal opening in Rio Grande tomato plants infected with P. syringae pv. tomato DC3000 (Pst) and Pst DC3000 containing deletions in genes avrPto and avrPtoB (Pst DC3000 ΔavrPto/ΔavrPtoB). Samples were collected 1 h, 4 h and 24 h after infection. Bars represent the stomatal aperture ratio mean values ± SD of three biological replicates of one representative experiment. Asterisk (*) and double asterisks (**) indicate significant differences between tomato plants infected with Pst DC3000 and those infected with Pst DC3000 ΔavrPto/ΔavrPtoB, with $p < 0.05$ and $p < 0.01$, respectively.
