# Supplementary material for: A New Role For Green Leaf Volatile Esters in Tomato Stomatal Defense Against Pseudomonas syringe pv. tomato
Source: Front Plant Sci. 2018 Dec 18;9:1855. doi: 10.3389/fpls.2018.01855 (PMC6305539; doi:10.3389/fpls.2018.01855)
Supplement: Supplementary file 2 [file Data_Sheet_2.PDF]

Supplemental table 1

Gene	Forward primer (5’-3’)	Reverse primer (5’-3’)
PR1	ACTCAAGTAGTCTGGCGCAACTCA	AGTAAGGACGTTGTCCGATCGAGT
P23	TTCGAGGTACGCAACAACTG	ATGCATTGATGACCCATGTTT
AAT1	TTGCCTGTATGGGAAAGACA	AGATTCCCACGCAATTTTTG
JAZ7	TTGCTATGGCTCGTAGAGCAACTC	TTTGCCAATGAACGCTTGACGACG
JAZ9	TTTGGAGCTCACTCTTATGCCTCC	AGCTCAGTACGATCGGAAACCACA
P5CS1	ACCTTAATCTGGAGGCTTGA	AATTATTTCACCCACCTGCC
RAB18	CCTGGGATGCATTGAACACC	CACGGGACACCATAACACAC
Actin	CTAGGGTGGGTTCGCAGGAGATGATGC	GTCTTTTTGACCCATACCCACCATCACAC

Table S1. Primer sequences used for quantitative RT-PCR analysis of the selected tomato genes.
